# Atypical diffuse large B-cell lymphoma, primary splenic lymphoma variant; a case report

**DOI:** 10.1016/j.ijscr.2023.108861

**Published:** 2023-09-22

**Authors:** Muzi Meng, Cesar A. Riera, Jorge Mosquera, Harsh R. Parikh, Ajit Singh

**Affiliations:** a General Surgery, BronxCare Health System, Bronx, NY, USA; b School of Medicine, American University of the Caribbean, Cupecoy, St. Maarten, the Netherlands; c School of Medicine, St. George's University, Grenada

**Keywords:** Primary splenic lymphoma, Diffuse large B-cell lymphoma, Splenectomy, Non-Hodgkin's lymphoma

## Abstract

**Introduction and importance:**

Primary splenic lymphoma (PSL) is characterized by lymphoma involvement confined to the spleen and hilar lymph nodes, without evidence of liver involvement or other sites. This condition is extremely uncommon, accounting for approximately 1 % of non- Hodgkin lymphomas (NHLs) and <2 % of all lymphomas. Diffuse large B-cell lymphoma (DLBCL) is the most common histological subtype of both PSLs and all NHLs. DLBCL encompasses an aggressive heterogeneous entity with distinct morphological variants.

**Case presentation:**

A 68 year-old gentleman presented to the office with a 10-month history of vague left sided upper abdominal pain. Clinical examination revealed a tender left upper quadrant, evidenced with splenomegaly on radiological evaluation. The patient proceeded with a splenectomy with subsequent pathological and immunohistochemical analysis, confirming a final diagnosis of germinal center type DLBCL.

**Clinical discussion:**

Primary splenic DLBCL is a rare variant of DLBCL, characterized by exclusive involvement of the spleen. It requires a comprehensive diagnostic evaluation to exclude lymphoma involvement in other organs and lymph nodes. Splenectomy followed by appropriate adjuvant therapy has been demonstrated as the definitive treatment strategy. This case report emphasizes the importance of considering primary splenic DLBCL as a differential diagnosis in patients presenting with splenomegaly and highlights the significance of multidisciplinary collaboration for accurate diagnosis and optimal management of this uncommon entity. Conclusion: Primary Splenic DLBCL, an exceptionally rare B-Cell neoplasm variant, requires precise diagnosis due to its unique splenic involvement. Splenectomy's efficacy, adjuvant therapy, multidisciplinary collaboration, and ongoing research are crucial for optimal management.

## Highlights

• Novel PSL insights: uncommon spleen-restricted lymphoma highlighted, <1 % of NHLs
• DLBCL commonality: most PSLs & NHLs exhibit diffuse large B-cell lymphoma histology.
• Comprehensive evaluation essential to exclude extra-splenic involvement
• Splenectomy + adjuvant therapy validated as definitive treatment
• Multidisciplinary teamwork crucial for accurate diagnosis and optimal management

## 1 Introduction

Non-Hodgkin's Lymphoma (NHL) has a greater predilection for extranodal involvement, in comparison to Hodgkin Lymphoma [1,2]. However, malignant lymphoma can originate from any lymphoid tissue, even the spleen. In rare circumstances, large B-cell lymphoma can originate from the spleen, complicating the diagnosis. In view of the vague clinical presentation, it is difficult to make a clear diagnosis. Histopathology followed by immunohistochemistry is crucial in clinching the diagnosis in such cases.

Primary splenic lymphoma (PSL) is a rare neoplasm of the spleen, reported to make up <2 % of all lymphomas [3,5,21] and 1 % of all NHLs [4]. According to previous literature, it is recommended that a diagnosis of PSL should be made only if the disease is limited to the spleen or involves hilar lymph nodes without any recurrence after splenectomy [3,4].

Diffuse large B-cell lymphoma (DLBCL) is the most common histological subtype of both PSLs and all NHLs. DLBCL encompasses an aggressive heterogeneous entity with distinct morphological variants [6]. It is commonly seen in older men but can occur in any age group and gender. The clinical presentation is nonspecific and can be easily confused with other benign conditions. Clinical symptoms include weight loss, abdominal pain, anemia, and elevated lactate dehydrogenase (LDH) levels [[4], [5], [6]]. In this report, we describe a case of primary splenic DLBCL and discuss the clinical characteristics, diagnostic analysis, clinical management, and definitive treatment for this condition. The work has been reported in line with the SCARE criteria [29].

## 2 Case presentation

A 68-year-old male presented to the Emergency Department (ED) with complaints of left upper quadrant pain lasting two months. The patient had comorbidities of hypertension and dyslipidemia. The patient denied any history of weight loss, bruising, fever, chills, nausea, vomiting, hematochezia, melena, or recent travel.

Physical examination revealed tenderness in the left upper quadrant, consistent with spleen-tenderness without distention, rebound, guarding, or rigidity. Lymph nodes were non-palpable, presenting no evidence of regional lymphadenopathy. Complete blood count demonstrated leukocytosis, with lymphocyte-predominance, 16.0 %, and elevated monocyte count, 0.9 k/μL and 9.4 %. The patient also presented with an elevated LDH of 215 units/L. All remaining laboratory markers were within normal limits, including CRP at 1.41. The patient tested negative for viral markers of HIV, EBV, HTLV-1, Hepatitis B and Hepatitis C.

**Fig. 1:**
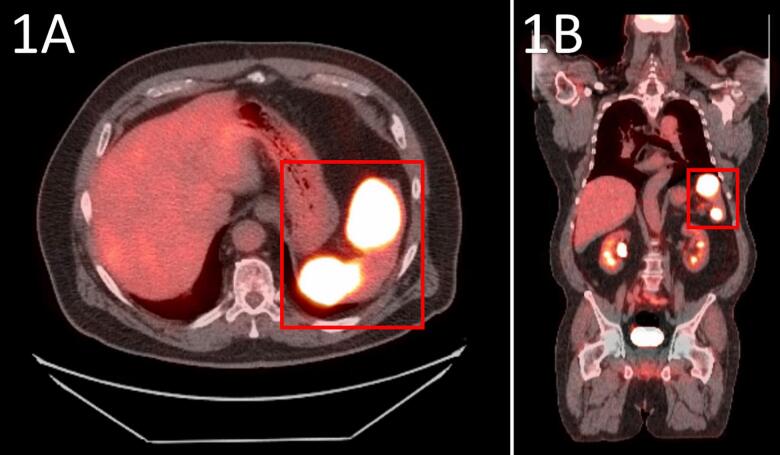
Axial (A) and coronal (B) PET/CT abdomen-pelvis scans demonstrating two hyper-attenuating lesions within the spleen, estimated at 4.1 × 2.5 cm and 1.5 × 3.0 cm. Hyper-attenuating PET scan images demonstrate areas of hypermetabolism, concerning for splenic malignancy, indicating definitive splenectomy and consequent pathology.

Ultrasound abdomen visualized a heterogeneous hypoechoic spleen, measuring 9.8 × 3.8 × 4.8 cm, demonstrating splenomegaly. Three well-circumscribed masses were identified on ultrasound. The first, a 3.6 × 3 × 3 cm in the mid-medical portion. The second, a 3.2 × 2.6 × 2.8 cm in the inferior portion of the spleen. The third, a 1.9 × 1.4 × 1.8 cm in the superior aspect of the spleen. PET-CT Abdomen-Pelvis revealed two hyper-attenuating lesions in the spleen, measuring 4.1 × 2.5 cm and 1.5 × 3.0 cm, consistent with hypermetabolic masses within the spleen (Fig. 1A & B). This constellation of radiological findings provided sufficient clinical indication for definitive Splenectomy with subsequent pathological analysis.

**Fig. 2:**
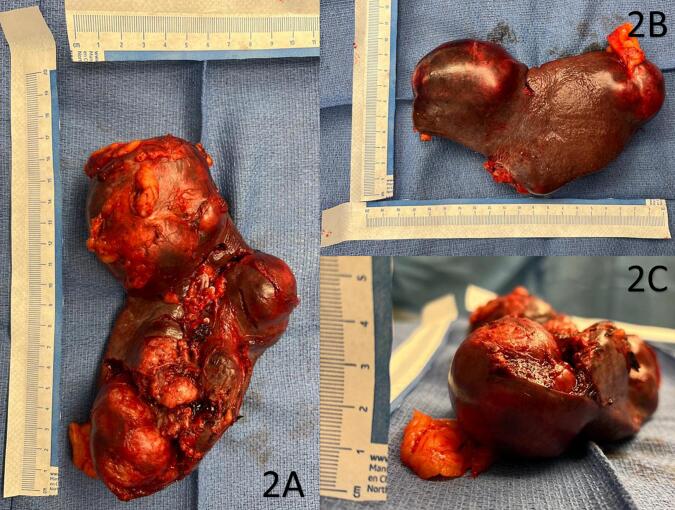
Resected spleen measuring 18 cm in length (A) and 9 cm in width (B) and 5 cm in depth (C). Gross examination of the specimen demonstrates significant splenomegaly, mass of 350 g, and diffuse distension of the splenic capsule with multiple subcapsular masses.

The patient underwent robotically-assisted laparoscopic splenectomy, overall resection of an 18 × 9 cm spleen and accessory spleen with hilum (Fig. 2A & B). Gross examination of the specimen revealed significant splenomegaly, weighing 350 g (Fig. 2A & C). The splenic capsule was significantly distended with multiple subcapsular masses (Fig. 2A & C). Two large masses were excised and sent for pathological immunohistochemical analysis.

Microscopic examination revealed well-delineated multinodular dense infiltrations of atypical lymphoid cells, with angulated rounded vesicular nuclei. Atypical lymphoid tissue was admixed with typical small lymphocytes, inconspicuous germinal centers, and lymphoid-infiltrating red pulp. Pathological findings were consistent with Lymphoma originating from the splenic-parenchyma. Immunohistochemical analysis of atypical lymphoid tissue was positive for surface markers CD20, CD21, and CD79a; negative for CD5, CD10, and CD23. Immunohistochemical gene analysis was positive for BCL6 dim, BCL2, HGAL, and LMO2; negative for MUM1, Cyclin D1, and SOX11. Ki-67 staining analysis identified a high mitotic proliferative index of atypical lymphoid tissue. Pathological and immunohistochemical analysis concluded a final diagnosis of diffuse large B-cell lymphoma (DLBCL), germinal center subtype, with surrounding typical splenic tissue and no evidence of capsular invasion and consequent metastasis.

The patient was discharged on post-operative day two, following vaccinations for *Haemophilus*, *Meningococcal Group B*, and *Neisseria meningitidis* for prophylaxis of capsular organisms, per recommendations for infectious diseases.

Outpatient post-operative visits were unremarkable with surgical scars healing well and no concerns for infection. The patient received an insertion of a central tunneled venous catheter for adjunct chemotherapy three months post-splenectomy. The patient underwent four cycles of R-CHOP adjuvant chemoradiation therapy: localized radiotherapy with Cyclophosphamide, Hydroxydaunorubicin (Adriamycin), Oncovin (Vincristine), Prednisone, and Rituximab. At six months post-splenectomy, the patient demonstrated no signs of cancer recurrence confirmed by the PET scan and is recovering well.

## 3 Discussion

Primary tumors of the spleen, both benign and malignant, are incredibly rare. Primary malignant lymphoma of the spleen (PMLS), also known as splenic lymphoma, is rarer still, making up <1 % of all non-Hodgkin lymphomas [8,9]. Primary splenic lymphomas have been associated with the hepatitis C virus and HIV infections [10]. Both viruses are thought to contribute to autoimmune disorders of the bone marrow B cells, and consequent malignant transformations [10]. Other subtypes of splenic lymphomas include splenic villous lymphoma, splenic marginal zone lymphoma (small B cell), and follicular lymphoma [11]. Epidemiologically, men are more frequently affected than women and it tends to present in late middle age or later; although it can occur at any age [9,12].

Diffuse large B-cell lymphoma (DLBCL) — the most common form of NHL in adults is one of the less prevalent types of splenic NHL [13]. The spleen may be involved as part of a systemic lymphoma or as the progenitor site for lymphoma; the latter is termed primary splenic diffuse large B-cell lymphoma (PS-DLBCL). Patients with DLBCL typically present with fever, fatigue, weight loss, chills, nausea, palpable splenomegaly, and possibly left upper quadrant pain or other nonspecific systemic symptoms [14,15]. Cytopenia is the most commonly seen laboratory abnormality, with anemia being the most common [16]. The first sonographic sign of DLBCL is a well-circumscribed, nodular mass within the spleen [8,14]. The mass is almost always hypoechoic. There may be associated splenomegaly or lymphadenopathy with possible surrounding areas of necrosis [15].

By immunohistochemistry, the tumor cells are positive for CD20, CD21, CD79a, and BCL6dim, while negative for CD5, CD10, CD23, MUM1, cyclin D1, and SOX11 with high Ki-67 in this patient, favouring a diagnosis of DLBCL. p53 and C-MYC stain scattered cells and EBER ISH was negative. The morphologic, immunophenotypic and cytogenetic findings were concluded to be diagnostic of DLBCL, Germinal center B cell (GCB) subtype using the Hans algorithm [28].

DLBCL is an aggressive cancer and is quick to spread. Therefore, early detection and timely intervention are critical for survival [12]. Splenectomy was recommended as a useful diagnostic and therapeutic tool by earlier researchers [13,23]. Additionally, it can provide symptomatic relief and reduce hypersplenism. The study done by Bairey et al. shows that splenectomy performed in early-stage PS-DLBCL has therapeutic impacts that significantly enhance the patient's condition and offer improved survival outcomes [7]. Robotic splenectomy was favoured over laparoscopic splenectomy since it minimise intra-op blood loss and post-op hospital stay, as recent studies comparing the two techniques for non-traumatic splenic pathologies [27].

In patients with multiple co-morbidities, advanced age and high risk of splenic rupture, splenectomy is not recommended. With advancements in less invasive techniques, such high-risk patients can benefit from fewer complications and effective diagnostic evidence provided by needle core biopsy, as shown by recent comparative studies [22,24].

Adjuvant chemoradiation therapy can be used in conjunction with surgery to eliminate any remaining cancerous cells. Adjuvant chemotherapy commonly consists of R-CHOP therapy: localized radiotherapy with Cyclophosphamide, Hydroxydaunorubicin (Adriamycin), Oncovin (Vincristine), and Prednisone. Additionally, a chimeric anti-CD20 antibody, Rituximab, is a common addition to R-CHOP therapy for primary B-cell neoplasms [10,12,16]. Rituximab and chemotherapy have been demonstrated to have a better outcome when used to treat diffuse large B cell lymphoma of the spleen, as shown by the longer survival of patients. According to a study, the three-year survival rates were 95 %, 100 %, and 55 %, respectively, while the overall response rates were 88 % with rituximab, 83 % with rituximab plus chemotherapy, and 55 % with chemotherapy alone [25].

The prognosis for the different subtypes of primary malignant splenic lymphoma depends on the stage of the cancer. Stage I is defined as malignancy being limited to the spleen, whereas stages II and III are characterized by hilar node involvement and distant metastasis, respectively [17]. Metastasis outside the spleen and involvement of the hilar nodes are the most critical factors when evaluating disease prognosis [17]. When the cancer is isolated to the spleen or its adjacent hilum, stages I or II, the prognosis is favorable, with a median survival of 7.48 years [18].

Although secondary involvement of the spleen is a common finding in nodal and extranodal lymphomas, primary lymphomas of the spleen represent an extremely rare pathology. A strict diagnostic criterion is essential to confirm the diagnosis. Criteria include lymphoma-associated splenomegaly, malignancy restriction to the spleen with no extranidal involvement, and absence of hepatomegaly [3].

Relapse is a common problem with those who suffer from DLBCL, as 40 % of those with DLBCL will eventually suffer from malignancy recurrence [19]. Relapse is diagnosed with whole body F-deoxyglucose (FDG) PET scan or biopsy [20]. Adjuvant chemoradiation therapy post-splenectomy can be utilized for prophylaxis against malignancy recurrence [10,12,16]. In their study of patients with primary splenic lymphoma, Brox et al. concluded that splenectomy alone or splenectomy combined with treatment resulted in a median survival time of 7.48 years. They noted no relationship between prognosis and histological subtype [6].

In circumstances where surgery is not an option, splenic irradiation may be used to reduce the size of the spleen. The International Prognostic Index (IPI), which predicts the overall and progression-free survival in patients with aggressive NHLs, was found to be significantly more accurate than the Ann Arbor classification for prognostication. One point is assigned for each of the following risk factors: age >60 years, stage III or IV disease, elevated serum LDH, ECOG/Zubrod performance status of 2, 3, or 4 and involvement of more than one extranodal site. The sum of the points allotted correlates with the following risk groups: low risk (0–1 points), low-intermediate risk (2 points), high-intermediate risk (3 points), and high risk (4–5 points) with a 3-year overall survival of 91 %, 81 %, 65 % respectively and a 5-year survival of 73 %, 51 %, 43 % and 26 % respectively [26].

The Oncology consultation concluded that this patient had DLBCL Stage 1 with extranodal involvement of the spleen, most likely to be Stage 1E (S). The IPI was calculated to be 1 point (Low risk), with age being the only factor on the positive side. Based on this, the recommended post-splenectomy plan was three cycles of chemotherapy followed by a PET scan and three cycles of R-CHOP followed by interim staging PET scan.

## 4 Conclusion

Primary splenic diffuse large B-cell lymphoma (DLBCL) is an exceedingly rare B-cell neoplasm variant, characterized by exclusive involvement of the spleen. Notwithstanding its rarity, it may be underreported. It requires a comprehensive diagnostic evaluation to exclude extranodal lymphoma involvement or associated lymphadenopathy. Splenectomy followed by appropriate adjuvant therapy has been demonstrated as an effective treatment strategy in these cases and prophylaxis for malignancy recurrence. This case report emphasizes the importance of considering primary splenic DLBCL as a differential diagnosis in patients presenting with splenomegaly and highlights the significance of multidisciplinary collaboration for accurate diagnosis and optimal management of this uncommon pathology. Future investigations should be directed toward establishing standardized diagnostic criteria, clinical management algorithms, and guidelines for long-term treatment management.

## Funding

N/A.

## Ethical approval

Ethics approval is not necessary since no novel treatment or detracting from standard treatment guidelines.

## Registration of research studies

N/A.

## Consent

Written informed consent was obtained from the patient for publication and any accompanying images. A copy of written consent is available for review by the Editor-in-Chief of this journal on request.

## Credit authorship contribution statement

Muzi Meng-Writing – original draft preparation; Writing – review & editing.

Cesar Riera- Writing – review & editing.

Jorge Mosquera- Writing – review & editing.

Harsh R Parikh- Writing – review & editing.

Ajit Singh- Attending Surgeon, Supervision.

Guarantor: Ajit Singh.

## Declaration of competing interest

N/A.
